# Commensal and Pathogenic Biofilms Alter Toll-Like Receptor Signaling in Reconstructed Human Gingiva

**DOI:** 10.3389/fcimb.2019.00282

**Published:** 2019-08-07

**Authors:** Lin Shang, Dongmei Deng, Jeroen Kees Buskermolen, Sanne Roffel, Marleen Marga Janus, Bastiaan Philip Krom, Wim Crielaard, Susan Gibbs

**Affiliations:** ^1^Department of Preventive Dentistry, Academic Centre for Dentistry Amsterdam (ACTA), University of Amsterdam and Vrije Universiteit Amsterdam, Amsterdam, Netherlands; ^2^Department of Oral Cell Biology, Academic Centre for Dentistry Amsterdam (ACTA), University of Amsterdam and Vrije Universiteit Amsterdam, Amsterdam, Netherlands; ^3^Department of Molecular Cell Biology and Immunology, Amsterdam UMC, Vrije Universiteit Amsterdam, Amsterdam, Netherlands

**Keywords:** TLR, gingiva, organotypic 3D culture, innate immunity, biofilm

## Abstract

The balance between the host and microbe is pivotal for oral health. A dysbiotic oral microbiome and the subsequent host inflammatory response are causes for the most common dental problems, such as periodontitis and caries. Classically, toll-like receptors (TLRs) are known to play important roles in host-microbe interactions by recognizing pathogens and activating innate immunity. However, emerging evidence suggests that commensals may also exploit TLRs to induce tolerance to the benefit of the host, especially in oral mucosa which is heavily colonized by abundant microbes. How TLRs and downstream signaling events are affected by different oral microbial communities to regulate host responses is still unknown. To compare such human host-microbe interactions *in vitro*, we exposed a reconstructed human gingiva (RHG) to commensal or pathogenic (gingivitis, cariogenic) multi-species oral biofilms cultured from human saliva. These biofilms contain *in vivo* like phylogenic numbers and typical bacterial genera. After 24 h biofilm exposure, TLR protein and gene expression of 84 TLR pathway related genes were investigated. Commensal and pathogenic biofilms differentially regulated TLR protein expression. Commensal biofilm up-regulated the transcription of a large group of key genes, which are involved in TLR signaling, including TLR7, the MyD88-dependent pathway (CD14, MyD88, TIRAP, TRAF6, IRAKs), MyD88-independent pathway (TAB1, TBK1, IRF3), and their downstream signaling pathways (NF-κB and MAPK pathways). In comparison, gingivitis biofilm activated fewer genes (e.g., TLR4) and cariogenic biofilm suppressed CD14, IRAK4, and IRF3 transcription. Fluorescence *in situ* hybridization staining showed the rRNA of the topically applied and invaded bacteria, and histology showed that the biofilms had no obvious detrimental effect on RHG morphology. These results show an important role of TLR signaling pathways in regulating host-microbe interactions: when a sterile gingival tissue is exposed to commensals, a strong immune activation occurs which may prime the host against potential challenges in order to maintain oral host-microbe homeostasis. In contrast, pathogenic biofilms stimulate a weaker immune response which might facilitate immune evasion thus enabling pathogens to penetrate undetected into the tissues.

## Introduction

Within the oral cavity, the dense commensal microbe environment maintains the gingiva in a mildly activated state so that it is able to respond directly to pathogenic challenges in order to maintain homeostasis (Devine et al., 2015). This homeostatic balance is controlled by continuous priming of the host via key receptors, known as toll-like receptors (TLRs) (Akira and Takeda, 2004). TLRs span the cell membrane and are able to activate downstream signaling pathways that ultimately influence the production of cytokines and defensins, cell proliferation, and survival (Hans and Hans, 2011; Kubinak and Round, 2012). Therefore, TLRs, by forming the first line of host defense, play a pivotal role in regulating innate immunity and further serve as a bridge between innate and adaptive immunity. Increasing evidence suggests that TLRs might be involved in guiding the host to respond appropriately to commensal as well as pathogen exposure (Hans and Hans, 2011; Kumar and Mason, 2015; Lamont et al., 2018).

Triggering of TLRs by pathogens is classically understood to result in an up-regulated inflammatory response that supports the subsequent clearance of the offending microbes (McClure and Massari, 2014). After the activation of TLRs, a number of downstream pathways are regulated, including nuclear factor kappa B (NF-κB), interferon regulatory factor (IRF) and mitogen-activated protein kinase (MAPK) pathways which target chemokine, cytokine and type I interferon production (Dowling and Mansell, 2016). However paradoxically, studies also suggest that activated TLRs regulate beneficial commensal-host interactions, such as promoting commensal colonization (Round et al., 2011), balancing inflammatory response, limiting bacteria penetration (Vaishnava et al., 2008), inducing tolerance in the host immune system, and establishing epithelial barrier function (Hooper and Macpherson, 2010; Kubinak and Round, 2012). For example, the colonization of intestinal commensal *Bacteroides fragilis* protects mice from developing experimental inflammatory bowel disease via TLR2 (Round and Mazmanian, 2010). The complete lack of TLR signaling in MyD88^−/−^ mice resulted in more severe intestinal inflammation than in wild-type mice (Rakoff-Nahoum et al., 2004). TLR3 activated by skin commensal *Staphylococcus epidermidis* was found to be involved in amplifying the innate immune response and inducing antimicrobial peptide expression in keratinocytes (Lai et al., 2009). In the oral cavity, TLRs are suggested to be engaged in building and maintaining host-microbe homeostasis. Activated by oral commensals, TLR signaling leads to production and activation of signal transduction proteins such as AP-1 (activator protein 1) and NF-κB, which induce expression of neutrophil chemoattractant CXCL2 and CXCL8 (Zenobia et al., 2013; Devine et al., 2015). Recruited neutrophils limit microbial invasion and thereby maintain oral health. Comparison of healthy and periodontitis tissues showed that periodontitis was characterized by loss of TLR1, TLR2, and TLR5 expression in the superficial epithelial cell layers (Beklen et al., 2008).

TLRs have been extensively studied with regards to host-microbe interactions (Chu and Mazmanian, 2013; Kawasaki and Kawai, 2014). However, little is known about the TLR signaling events when a sterile healthy tissue is exposed to a microbial community for the first time. Previous studies mainly focused on either the function of one TLR (Hans and Hans, 2011; Dowling and Mansell, 2016), or the reaction of different TLRs to one single microbial species in simple cell culture assays (McClure and Massari, 2014). Some studies used animal models in spite of the species-specific expression of TLRs (Rehli, 2002). Others have focused on clinical studies describing the TLR expression in the diseased tissue exposed to oral microbiome (Beklen et al., 2008). However, both animal and clinical studies have ethical and scalability limitations. To study host-microbe interactions in a more physiological setting, whilst at the same time developing a novel *in vitro* alternative to the use of animals for scientific purposes, we combined two state of the art models: 3D organotypic reconstructed human gingiva model (RHG) and multi-species biofilms (Buskermolen et al., 2016, 2017). The RHG showed many characteristics similar to native gingiva. Morphologically, it consists of a multi-layered epithelium on a fibroblast-populated hydrogel which functions as a lamina propria. RHG secretes host protective molecules (e.g., cytokines and antimicrobial peptide Elafin) and the epithelium expresses typical keratins characteristic of differentiated gingiva (e.g., high keratin 13 and keratin 17) as found *in vivo* (Kosten et al., 2015; Buskermolen et al., 2016). The multi-species biofilms were grown under defined conditions from human saliva to represent three distinct phenotypes: commensal, gingivitis, and cariogenic. These biofilms each contain physiological numbers and typical bacterial genera, and are metabolically active (lactic acid and protease production) in line with the phenotypical differences of the *in vivo* biofilms, and influenced RHG cytokine secretion (Janus et al., 2015; Buskermolen et al., 2017). Furthermore, the commensal biofilm showed a long-term beneficial influence on the RHG, by enhancing proliferation and stratification of the epithelium barrier and inducing secretion of antimicrobial peptide, cytokines and chemokines to combat potential external challenge (Buskermolen et al., 2017; Shang et al., 2018). Pathogenic biofilms in contrast failed to trigger such an efficient host response facilitating immune evasion (Buskermolen et al., 2017). The cellular mechanisms and TLR signaling pathways that result in these different host responses to commensals and pathogens are currently unknown.

The aim of this study was to investigate how commensal and pathogenic biofilms influence TLR signaling in a sterile RHG model. RHG were exposed to different biofilms (commensal, gingivitis, or cariogenic), and their influence on RHG (i) histology; (ii) expression of genes involved in TLR signaling pathways; and (iii) TLR protein expression was determined.

## Materials and Methods

### Multi-Species Biofilm

Human saliva collected from 10 healthy donors was pooled and used as inoculum for multi-species biofilm as previously described (Janus et al., 2015). The 10 healthy donors had no complaints which required treatment by a dental specialist. The saliva was collected following the ethical principles of the 64th World Medical Association Declaration of Helsinki, and the procedure was approved by the institutional review board of the Amsterdam UMC (Amsterdam, The Netherlands). The biofilms were cultured from the healthy saliva in the Amsterdam active attachment model (AAA-model) anaerobically, by using three different types of medium to promote the growth of commensal, gingivitis or cariogenic biofilms as previously described (Janus et al., 2015; Buskermolen et al., 2017). In short, a semi-defined McBain medium was used as basic culture medium (for commensal biofilm) and extra supplements were added to promote the transition of the pathogenic biofilms: 10% fetal calf serum (FCS) for gingivitis biofilm or 0.2% sucrose for cariogenic biofilm. In line with the disease phenotypes, the caries phenotype was confirmed by higher lactic acid production in a colorimetric assay and the gingivitis phenotype was confirmed by an overall higher protease activity in a fluorescence resonance energy transfer (FRET) assay as previously published (Janus et al., 2015). The anaerobic colony forming units (CFU) of all the biofilms were assessed by viable bacterial cell counting. After culturing, the aliquots of the biofilms were frozen at −80°C until use.

### RHG Culture

Immortalized human gingiva cell lines (keratinocytes and fibroblasts) were cultured and used to construct RHG exactly as described previously (Buskermolen et al., 2016): keratinocyte (KC-TERT, OKG4/bmi1/TERT, Rheinwald laboratory, Boston, MA, USA) (Dickson et al., 2000) and fibroblast (Fib-TERT, T0026, ABM, Richmond, BC, Canada). In short, a collagen solution containing fibroblasts was pipetted into a six-well transwell insert with 0.4 μm pores (Corning). After 3 days culture, keratinocytes (5 × 10^5^ cells/transwell) were pipetted on top of the fibroblast-populated collagen hydrogel. After a further 3 days submerged culture, RHG was lifted at the air—liquid interface and further cultured for 10 days in which time a differentiated, stratified epithelium developed on the fibroblast-populated collagen hydrogel.

### Exposure of RHG to Biofilm

The stored biofilms were thawed on ice, centrifuged and dispersed in Hanks' balanced salt solution (HBSS, Sigma-Aldrich) (Spano et al., 2016). A sample of biofilm was processed to determine CFU count. The remaining biofilm was applied topically onto RHG, with ~1 × 10^7^ CFU biofilm cells concentrated in a drop of 10 μl in HBSS as previously described (Buskermolen et al., 2017). The biofilm-RHGs were further cultured at the air liquid interface in medium containing DMEM/Ham's F12 (3/1) (Gibco, Grand Island, USA) supplemented with 1% Fetal Clone III (RHG, Logan, UT, USA), 0.1 μM insulin (Sigma-Aldrich, St. Louis, MO, USA), 2 μM hydrocortisone (Sigma-Aldrich), 1 μM isoproterenol (Sigma-Aldrich), 10 μM carnitine (Sigma-Aldrich), 10 mM L-serine (Sigma-Aldrich), 0.4 mM L-ascorbic acid (Sigma-Aldrich), and 2 ng/ml epidermal growth factor (Sigma-Aldrich), for 24 h at 37°C, 7.5% CO_2_ and 95% humidity. After 24-h exposure RHG were harvested.

### Fluorescence *in situ* Hybridization and Histological Staining

RHGs were harvested, fixed in 4% paraformaldehyde and processed for paraffin embedment. Embedded tissues were cut into 5 μm sections for staining. Fluorescence *in situ* hybridization (FISH, probe 10-ME-H000, BioVisible), hematoxylin and eosin (H&E) staining were performed as previously described (Buskermolen et al., 2017). The images were taken using a fluorescence microscope (Nikon Eclipse 80i microscope with Nikon Plan Fluor 20x/0.50 and 40x/0.75 objectives) with NIS-Elements software (Nikon Instruments Europe B.V.).

### RNA and Protein Isolation

RHG epithelium was carefully removed with forceps from the fibroblast-distributed collagen hydrogel and washed with PBS once. Thereafter, total RNA and protein were isolated from the epithelium using a AllPrep RNA/Protein Kit (Qiagen, Hilden, Germany) following the manufacturer's instructions. Isolated RNA and protein were stored at −80°C before further processed for analysis.

### RT^2^ Profiler PCR Array

Genomic DNA was eliminated from RNA samples and cDNA synthesis was performed using RT^2^ First Strand Kit (Qiagen) according to the instructions supplied with the kit. cDNA was mixed with RT^2^ SYBR Green Master Mix (Qiagen) and processed for RT^2^ Profiler PCR Array (Human Toll-Like Receptor Signaling Pathway, PAHS-018Z, Qiagen). cDNA synthesis and the RT^2^ Profiler PCR Array were performed using Bio-Rad iCycler system (Bio-Rad, California, United States). Data was analyzed by the web-based software RT^2^ Profiler PCR Array Data Analysis using the ΔCt method (2^−ΔΔ*Ct*^) (Qiagen). All data were normalized to an average of housekeeping genes B2M and GAPDH. Data that did not meet the assumption for homogeneous variance of each of the three independent experiments were not analyzed further. A comparative heatmap was generated by the software showing the expression of individual gene normalized to the average expression of all the genes in the array. Genes were clustered based on the relationship of each gene to the average pooled expression of the array. The red color in **Figure 3A** represents up-regulated genes and green represents down-regulated genes, respectively.

### Western Blotting

Isolated protein was precipitated with ice-cold methanol for 15 min. After centrifugation, the protein pellet was collected and resolved in Complete Lysis-M buffer (Sigma-Aldrich, Missouri, United States). The amount of protein in each sample was measured using a Pierce BCA Protein Assay Kit (Thermo Scientific, Massachusetts, United States). After heating in Bolt LDS Sample Buffer (Invitrogen, California, United States) and Bolt Sample Reducing Agent (Invitrogen) at 95°C for 5 min, same amount of each protein sample (7 μg) were loaded and separated on 4–12% Bis-Tris Plus Gel (Invitrogen) and transferred to a nitrocellulose membrane (iBlot® 2 Transfer Stacks, Invitrogen). Membranes were blocked with 2% BSA PBST for 1 h and incubated with antibodies against TLR1, 2, 3, 5, 7, 8, 9 (1:1000, Novus Biologicals, Colorado, United States), TLR4 (1:200, Santa Cruz Biotechnology, Texas, United States) or β-actin (1:10000, Sigma- Aldrich) overnight at 4°C. Thereafter, membranes were washed three times in PBST and further incubated with Infrared Dye-conjugated secondary antibodies against mouse (1:7500 for TLR3, 4, 8, 9, and β-actin) or against rabbit (1:7500 for TLR1, 2, 5, 7). After washing, the blots were visualized using LI-COR Odyssey Scanner according to the manufacturer's instruction.

### Statistics

To calculate the differences between the RHG groups, a one-way ANOVA followed by Bonferroni's multiple comparison was performed. Differences were considered significant when *p* < 0.05. Data are represented as mean ± standard deviation; ^*^ = *p* < 0.05; ^**^ = *p* < 0.01; ^***^ = *p* < 0.001; ^****^ = *p* < 0.0001.

## Results

### Commensal and Pathogenic Biofilms Differentially Regulate TLR Signaling Pathways in RHG

**Figure 1:**
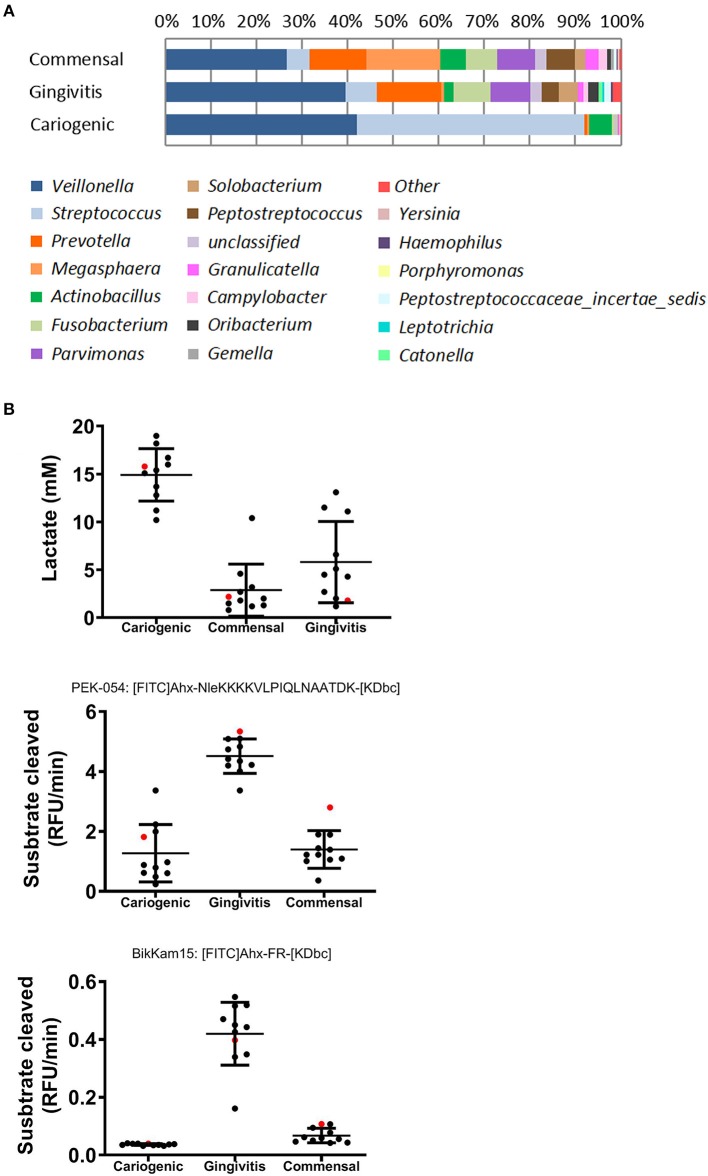
Composition and metabolites of commensal, gingivitis, and cariogenic biofilms. **(A)** Relative abundance of major bacterial genera of the three biofilms is shown. Remaining genera are included in “other”. **(B)** Lactic acid production, total protease activity (FRET probe: PEK-054) and gingipain protease activity (FRET probe: BikKam15) of biofilms that were cultured from saliva of individual donor (in black) or pooled saliva (in red). The same biofilms were used in this study as previously used in Janus et al. (2015) and Buskermolen et al. (2017).

The major genera of the commensal, gingivitis and cariogenic biofilms is shown in Figure 1A. Typical biomarkers for commensal (*Granulicatella*), gingivitis (*Catonella and Prevotella*), and cariogenic (*Streptococcus*) were present and corresponded to the phenotypes (Kumar et al., 2005; Zaura et al., 2009; Sanz et al., 2017). For a more extensive description of the biofilms, including Shannon diversity index and principle component analysis based on their operational taxonomic units, we refer you to (Buskermolen et al., 2017). For the metabolic products which are typically pathogenic for caries (lactic acid production) or for periodontitis (protease activity, shown as substrate cleaved speed), results of the three biofilms are cited from our previous study (Janus et al., 2015; Figure 1B).

**Figure 2:**
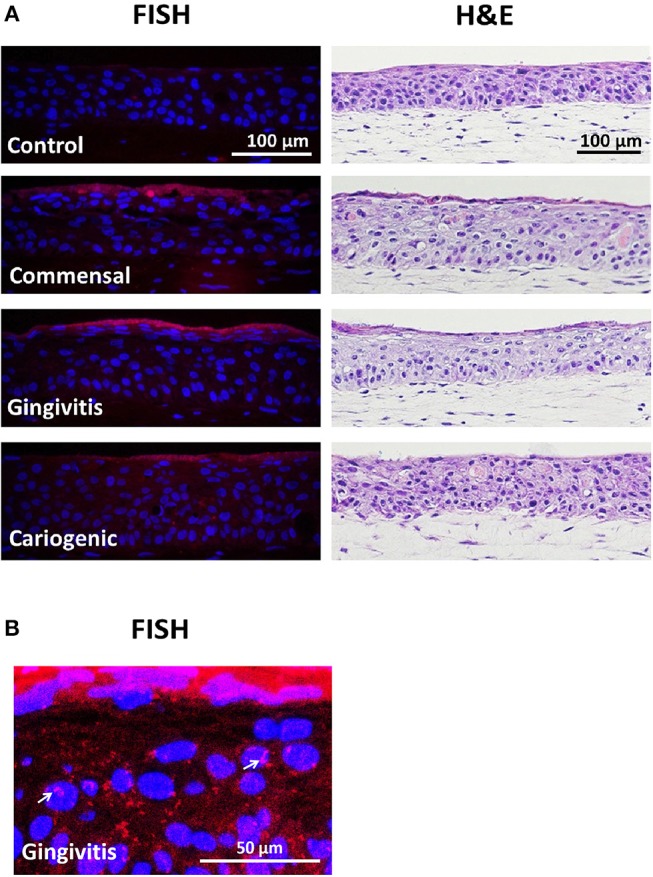
Biofilm exposure does not detrimentally influence RHG histology. **(A)** Biofilms were topically exposed to RHG for 24 h. Paraffin embedded tissue sections were used to visualize biofilm by fluorescence *in situ* hybridization (FISH, left panel) and RHG histology by hematoxylin and eosin staining (H&E, right panel). FISH shows a dense layer of bacterial rRNA (red) on top of the biofilm-exposed RHG, while no red was found in the unexposed RHG. The nuclei of epithelial keratinocytes in RHG are stained with DAPI (blue). H&E shows a stratified gingiva epithelium on a fibroblast populated collagen hydrogel. Data represent three independent experiments, each with an intra-experiment duplicate. **(B)** Enlarged FISH staining shows bacteria invading into RHG epithelium after gingivitis biofilm exposure (white arrows).

**Figure 3:**
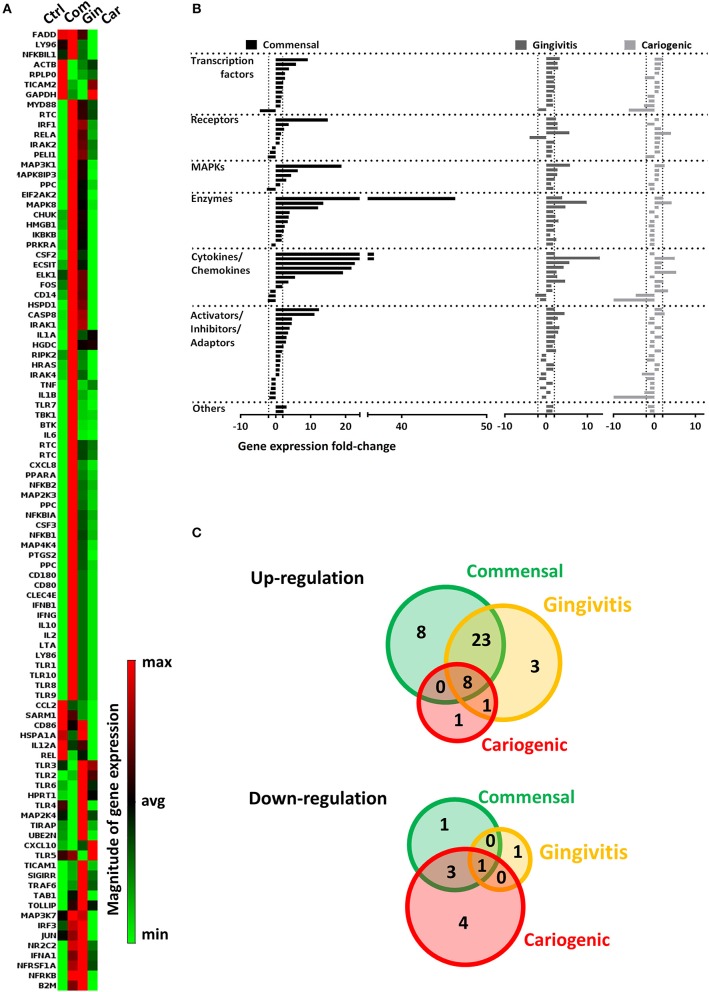
Commensal and pathogenic biofilms differentially regulate the transcription of TLR signaling pathway in RHG. After 24 h exposure, mRNA expression of 84 key genes involved in TLR signaling was investigated using a preformatted RT^2^ Profiler PCR array. Data which were not homogenous in each of the three independent experiments were excluded from further analysis. **(A)** The heatmap shows the regulated expression of the genes in control (unexposed RHG), commensal, gingivitis, and cariogenic biofilm exposed RHG. The magnitude of each individual gene was determined by normalizing the 2^−ΔCT^ value to the average 2^−ΔCT^ of all genes across the array, red shows high expression and green shows low expression. **(B)** The genes were further grouped based on their biological function and are shown by their fold regulation compared to the unexposed group. The vertical dashed lines indicate ± 2-fold regulation. **(C)** The diagrams show distribution and overlapping of genes that are either up-regulated or down-regulated by biofilm exposure.

To investigate the influence on RHG TLR signaling, RHGs were topically exposed to commensal, gingivitis or cariogenic biofilms (Figure 2). Already within the 24 h exposure period, biofilm RHG showed a thickened epithelium on the fibroblast populated collagen hydrogel compared to unexposed RHG (exposed to HBSS medium without biofilm) in line with our previous findings (Shang et al., 2018). FISH rRNA staining showed a compact bacterial layer on the surface of all the biofilm exposed RHG and sporadic bacteria invading the viable layers of the epithelium (Figures 2A,B). The TLR signaling pathway transcription was investigated using a preformatted qPCR array, and genes were excluded which did not meet the assumption of homogenous variance by the qPCR cycles among each of the three independent experiments (Figure 3). The heatmap shows differential expression of 84 TLR pathway related genes in the epithelium after exposure to the different biofilms when compared to unexposed control RHG (Figure 3A). Compared to the average expression of all the investigated genes, commensal biofilm had the highest expression of many genes (most red compared to control RHG). Gingivitis biofilm clearly had a high expression of a different group of genes and cariogenic biofilm showed less but still distinct effects. Genes were further grouped according to their biological function (Figure 3B). Compared to unexposed RHG, commensal and gingivitis biofilms increased expression of many genes from all the listed function groups, whereas cariogenic biofilm only up-regulated expression of a few genes related to receptors, enzymes, cytokines and chemokines (Figure 3B and Supplementary 1). From the 84 investigated genes, commensal biofilm increased the expression of 39 genes, including 12 genes > 10-fold, 4 genes > 5-fold, and 23 genes > 2-fold compared to the control group (Figure 3C and Table 1). Gingivitis biofilm up-regulated 35 genes, with 1 gene > 10-fold, 4 genes > 5-fold, and 30 genes > 2-fold. The cariogenic biofilm group had the least up-regulating effect on the TLR-related genes (10 genes in total, 1 gene > 5-fold and 9 genes > 2-fold) but the most significant down-regulation (the number of genes down-regulated by commensal: 5 genes > 2-fold; gingivitis: 2 genes > 2-fold; cariogenic: 2 genes > 10-fold, 1 gene > 5-fold and 5 genes > 2-fold). Commensal and gingivitis biofilm up-regulated 31 TLR-related genes in common, which are involved in multiple functions (Figure 3C and Supplementary 1). Of the 10 genes that were up-regulated by cariogenic biofilm, eight can be increased by all the biofilms, indicating a less specific effect of the cariogenic biofilm on promoting TLR pathways.

**Table 1:** Genes up/down-regulated more than 2-fold.

**Gene/Group**	**Commensal**	**Gingivitis**	**Cariogenic**
BTK	46.31	3.91	2.12
CSF3	36.76	13.00	4.92
IL6	36.76	2.09	–
CXCL8	22.63	5.66	–
IL1B	21.61	4.29	–
TNF	19.25	2.58	5.28
MAP2K3	18.81	5.79	2.52
TLR7	14.93	2.41	–
IRAK2	13.61	9.85	4.19
TBK1	12.41	2.09	–
PTGS2	12.13	4.70	–
NFKBIA	11.06	4.49	2.46
NFKB2	9.19	3.33	2.05
MAP4K4	6.35	2.76	–
NFKB1	5.79	2.76	–
CSF2	5.53	2.70	–
IKBKB	4.70	2.83	–
HRAS	4.59	–	–
MAPK8	4.39	2.70	–
PELI1	4.00	3.25	–
IRAK4	4.00	–	–
RELA	3.82	2.89	–
CD14	3.73	2.76	–
EIF2AK2	3.73	2.30	–
IFNA1	3.65	4.59	2.17
HSPD1	3.56	2.89	–
CASP8	3.48	3.03	–
MYD88	3.10	2.24	–
ECSIT	3.10	–	–
MAP3K1	3.03	2.14	–
MAPK8IP3	2.96	2.09	–
PPARA	2.70	–	–
CHUK	2.58	–	–
TNFRSF1A	2.46	2.76	–
IRAK1	2.30	2.09	–
HMGB1	2.24	–	–
PRKRA	2.19	–	–
IRF1	2.14	–	–
NR2C2	2.09	2.19	–
TLR2	–	5.66	4.05
TAB1	–	2.52	–
CXCL10	–	–	3.29
TOLLIP	–	2.41	–
SIGIRR	–	2.05	–
FOS	2.70	2.05	−2.35
CCL2	−2.09	−2.64	−4.54
TLR4	−2.24	–	−2.19
IL12A	−2.24	–	−10.56
REL	−4.49	–	−6.28
MAP2K4	−2.52	–	–
JUN	–	–	−2.55
TLR5	–	−4.00	–
FADD	–	–	−3.07
HSPA1A	–	–	−2.46
CD86	–	–	−12.55

Regarding down-regulated genes (Figure 3C and Table 1), cariogenic biofilm uniquely down-regulated three genes which belonged to the functional group of activators, inhibitors, and adaptors (FADD, HSPA1A, CD86). Notably, MAP2K4 from the MAPK pathway was down-regulated (>2-fold) specifically by the commensal biofilm, and the immune stimulatory molecule IL12A was down-regulated >10-fold by the cariogenic biofilm (compared to 2-fold down-regulation by commensal biofilm and no regulation by gingivitis biofilm). Among all of the 55 regulated genes (>2-fold, both up and down regulated), FOS is the only one that was up-regulated by commensal and gingivitis biofilms (2-fold) but down-regulated by cariogenic biofilm (2-fold).

### Differential TLR Protein and mRNA Expression

**Figure 4:**
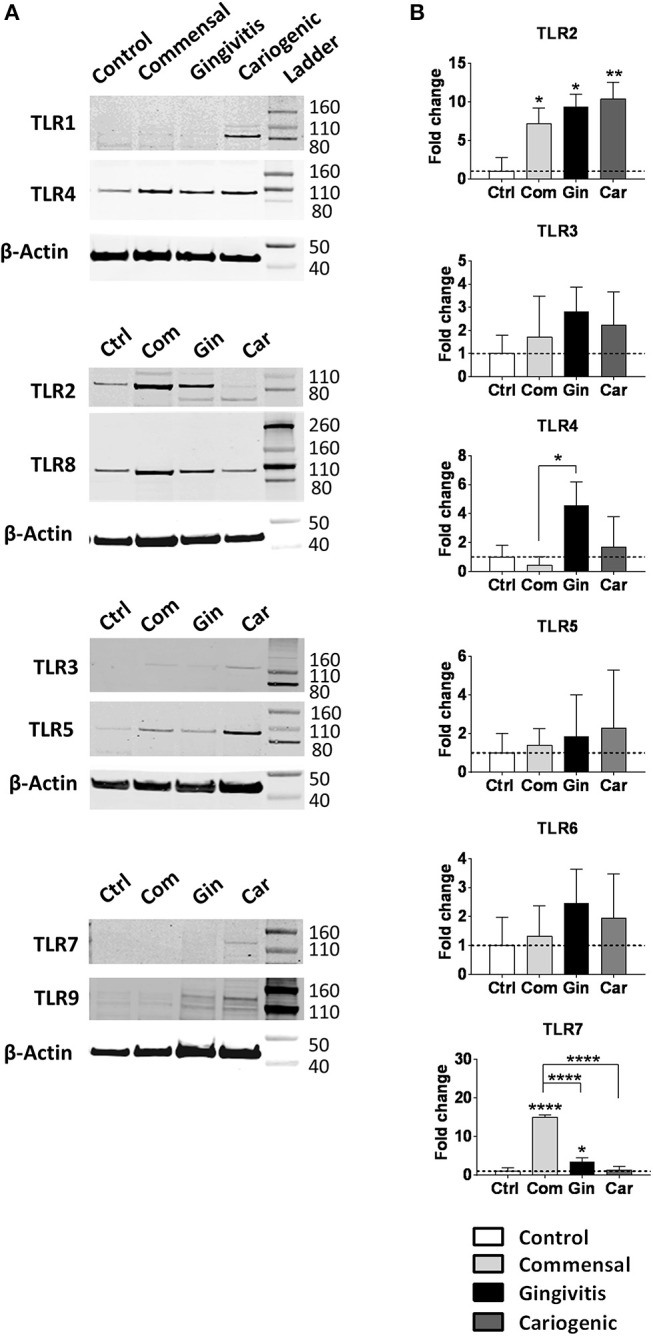
Commensal, gingivitis, and cariogenic biofilms regulate TLR expression differently. **(A)** Western blot analysis of TLRs 1-5 and 7-9 indicate the presence or absence of TLR proteins in RHG epithelium after 24 h biofilm exposure. **(B)** The TLR 2-7 mRNA expression after biofilm exposure. Data represent the average of three independent experiments (each with an intra-experiment duplicate) ± SD; ^*^p < 0.05; ^**^p < 0.01; ^****^p < 0.0001; one-way ANOVA followed by Bonferroni's multiple comparison for comparison between groups.

Next, epithelial TLR protein expression was investigated after the exposure to different biofilms (Figure 4A). Protein expression of TLRs 4, 5, and 8 was detected in all RHGs independent of whether they were exposed to biofilm or not. TLRS 1, 7, and 9 were only detected after pathogenic biofilm exposure (gingivitis and cariogenic) and TLR3 was weakly expressed after exposure to all three biofilms but not in unexposed RHG.

Since biofilm exposure was only for 24 h, which may not be sufficient to alter protein expression compared to the unexposed RHG, we next compared TLRs mRNA expression (Figure 4B). Of note, whilst protein was detected for TLR1 in cariogenic biofilm exposed RHG the mRNA level was below the 40 cycles threshold detectable for qPCR. TLR9 protein was clearly observed in gingivitis and cariogenic biofilms but again mRNA levels were below detection level. For TLR3 and TLR5, protein and mRNA levels were comparable and with no significant differences between the groups. TLR7 protein was barely observed in commensal biofilm exposed RHG but its mRNA expression showed the most significant increase (15-fold) in our study. In brief, all three types of biofilm strongly up-regulated TLR2 mRNA expression; TLR7 was significantly activated by commensal and gingivitis biofilms; TLR4 expression was only clearly increased by gingivitis biofilm; TLRs 3, 5, and 6 were not significantly affected by the biofilms. The mRNA expression of TLRs 1, 8, 9, and 10 were regarded undetectable (Data not shown).

### Regulation of MyD88-Dependent and MyD88-Independent Pathways

**Figure 5:**
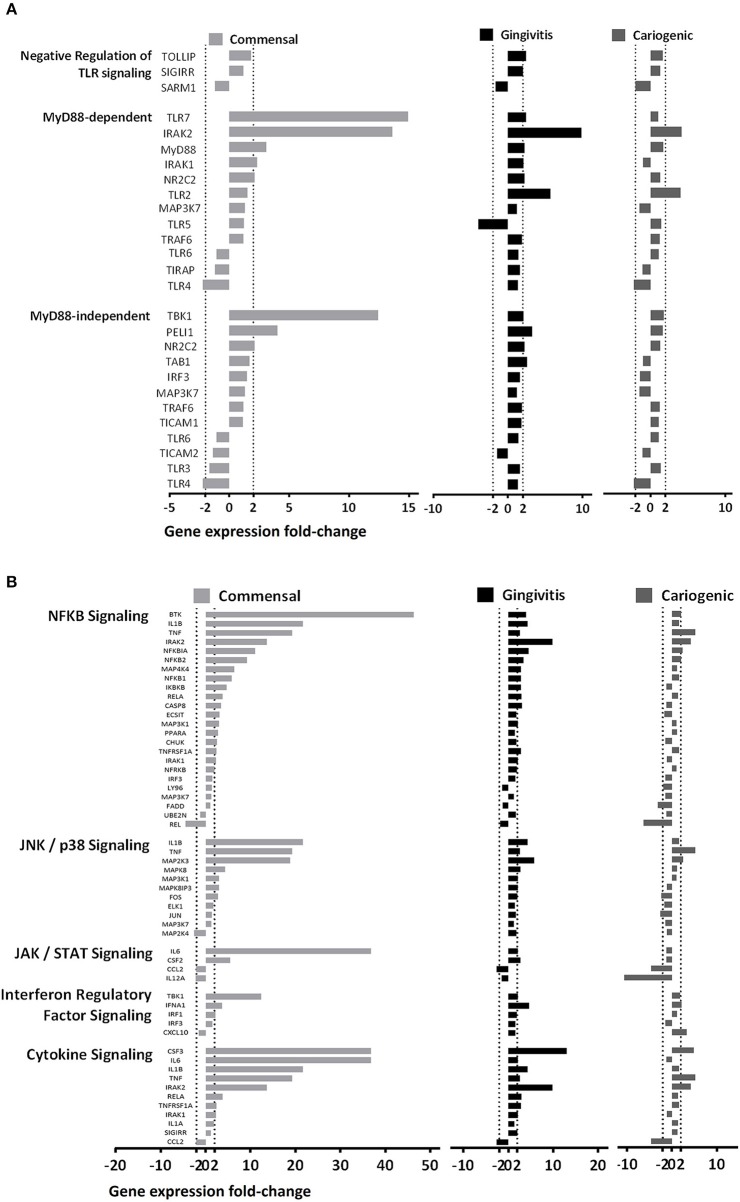
Biofilms differentially regulate MyD88 dependent and independent pathways. **(A)** Genes related to the two main TLR downstream cascades (MyD88-dependent and Myd88-independent pathways) are shown. The commensal biofilm up-regulated both pathways more than the gingivitis or the cariogenic biofilms. **(B)** Other downstream pathways related to TLR signaling are shown. The commensal biofilm showed strong up-regulation on all five pathways in comparison to the two pathogenic biofilms. Data represent three independent experiments, each with an intra-experiment duplicate.

In order to investigate in more detail how TLR downstream signaling is affected by the biofilms, we next compared the gene expression within the two major cascades, the MyD88-dependent and MyD88-independent pathways (Figure 5A). In general, genes from both pathways were broadly up-regulated by commensal biofilm and to a lesser extent by gingivitis biofilm whereas cariogenic biofilm only up-regulated two genes in the MyD88-dependent pathway. MyD88-dependent pathway was more activated than MyD88-independent pathway by commensal biofilm stimulation. No genes in MyD88-dependent or MyD88-independent pathways were down-regulated more than 5-fold by any of the biofilm exposed RHG.

Next, we grouped the genes according to five TLR downstream signaling pathways (Figure 5B). Generally, commensal biofilm significantly up-regulated all five pathways (NF-κB, JNK/p38, JAK/STAT, Interferon regulatory factor and cytokine signaling). Gingivitis biofilm also activated multiple genes of the pathways but to a lesser extent. Cariogenic biofilm showed very little up-regulation, but comparatively stronger downregulation on the JAK/STAT signaling pathway.

**Figure 6:**
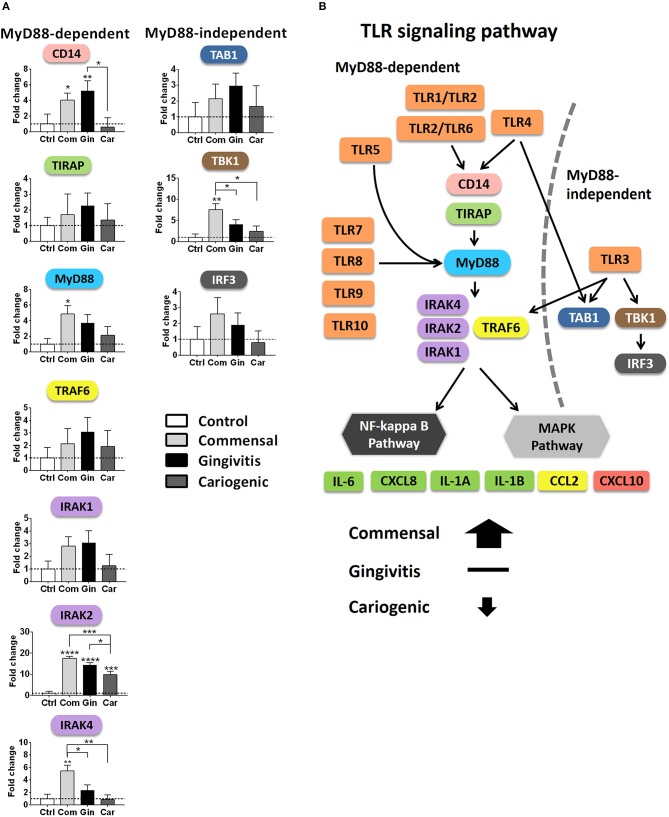
Overview of TLR signaling pathways after biofilm exposure. **(A)** The mRNA expression of key genes involved in the MyD88-dependent and independent pathways are shown after biofilm exposure. Data represent the average of three independent experiments, each with an intra-experiment duplicate ± SD; ^*^p < 0.05; ^**^p < 0.01; ^***^p < 0.001; ^****^p < 0.0001; one-way ANOVA followed by Bonferroni's multiple comparison for comparison between groups. **(B)** Diagram showing TLR signaling pathways summarizing the differential regulation by commensal and pathogenic (gingivitis, cariogenic) biofilm exposure.

Finally, we compared the expression of key genes individually, which are essential in driving downstream signaling (Figure 6A). MyD88, IRAK2, IRAK4 (MyD88-dependent), and TBK1 (MyD88-independent) were significantly up-regulated in RHG exposed to commensal biofilm compared to gingivitis or cariogenic biofilms. Gingivitis and cariogenic biofilms had little effect on both pathways except for gingivitis biofilm increasing CD14 and both biofilms increasing IRAK2 significantly.

## Discussion

Here we describe differential TLR activation in an organotypic human gingiva model after exposure to commensal and pathogenic multi-species oral biofilms. By exposing sterile RHG to either commensal or pathogenic biofilms we could investigate the immediate first response of the host tissue to a microbiome and demonstrate that a delicate balance exists which controls the strength and type of the host immune response. After commensal biofilm exposure, a strong immune activation occurs that may prime the tissue against potential assault by pathogens. When sterile RHG is exposed to pathogenic biofilms, a weaker immune response was found where multiple genes are suppressed and those that are activated are only done so moderately. Such a response would be expected to enable bacteria to invade the host tissue and cause tissue damage, e.g., periodontitis, if not sequestered by infiltrating immune cells.

In the oral cavity, biofilms continuously adapt to their different habitats. For example in the gingival sulcus, there are three types of biofilms that may diverge from each other and in doing so influence the periodontal tissues differently: (1) biofilm that attaches to the tooth surface (potential cariogenic), (2) biofilm that may cause inflammation in periodontal tissue or further invade into the soft tissues (gingivitis-periodontitis), and (3) biofilm that shows little harm or even beneficial effects on the host (commensal) (Moutsopoulos and Konkel, 2018). The gingivitis and cariogenic biofilms in this study were designed and cultured in a way to represent transitions from the original oral microbiome (commensal biofilm) to pathogenic biofilms. Cariogenic biofilm was included in this study since in root caries and caries developed on the proximal or cervical tooth surfaces, cariogenic biofilm is suspected to stimulate the interproximal papilla, attached gingiva, and marginal gingiva. However, in our study, the cariogenic biofilm resulted in less inflammatory response in RHG compared to the other two biofilms, which could indeed be due to the cariogenic biofilm being more pathogenic on tooth (hard tissue) than on gingiva (soft tissue).

The differential immune activation after multi-species biofilm exposure may be correlated to the diversity of the microbial community. A decreased diversity of oral microbiome *in vivo* is closely related with the development of dental caries (Gross et al., 2010), as well as with other imbalanced host responses (Verma et al., 2018). In line with this, we found that the RHG model responded differently to biofilms of different diversity via TLR signaling regulation: the commensal biofilm containing a higher species richness (70 ± 11 OTUs; Buskermolen et al., 2017) showed more up-regulation, while the cariogenic biofilm which is predominated by only two major genera (*Streptococcus* and *Veilonella*, 62 ± 8 OTUs) had the least activation with even a slight suppression of the host response occurring. Looking further at the genus level, *Streptococcus* and *Actinobacillus* were previously shown to activate TLR2-dependent signaling (Kikkert et al., 2007; Tomlinson et al., 2014). This is in line with our results showing that all the three biofilms contain *Streptococcus* and *Actinobacillus* in common and all up-regulated TLR2 transcription. Furthermore, the activation of TLR2 and TLR4 are found to be related with gingivitis and periodontitis *in vivo* (Yoshioka et al., 2008; Ilango et al., 2016), in line with our study where we found that the gingivitis biofilm induced more upregulation of TLR4 transcription compared to commensal and cariogenic biofilms.

When comparing TLR mRNA and protein expression, a number of differences were observed. TLR1, 8, and 9 mRNA were under the qPCR detection threshold whereas protein was clearly detectable. Since the TLR protein was already available to bind ligand, one can assume that mRNA transcription is therefore not required. In contrast, mRNA expression of TLR7 was high despite low protein expression. This would indicate that transcription and translation was occurring to present TLR7 on the host intracellular membrane particularly in the case of commensal biofilm exposure.

Next to the TLRs, our results show that commensal and pathogenic oral biofilms differentially activate the downstream signaling cascades in RHG: The MyD88-dependent and independent, as well as the following NF-κB and MAPKs pathways are visualized in Figure 6B. Both the MyD88-dependent and MyD88-independent pathways were activated by commensal biofilm via transcriptional up-regulation of key adaptor proteins CD14, MyD88, TIRAP, TRAF6, IRAKs (MyD88-dependent), and TAB1, TBK1, IRF3 (MyD88-independent). In our previous study, protective cytokine secretion (IL-6, CXCL8, CCL20, and CCL2) was found to be significantly induced by the same commensal biofilm in RHG (Buskermolen et al., 2017), in line with the upstream activation of TLR signaling pathways described in this study. Among these cytokines, IL-6 has been shown to activate TLRs in mononuclear cells resulting in CXCL8, CCL2, and TNF-α secretion thus further amplifying the cytokine cascade (Caiello et al., 2014). It was also shown that single commensal species benefit the host by exploiting both MyD88-dependent pathway in oral epithelial cells, T cells and in periodontal tissue of mice (Zenobia et al., 2013; Wang et al., 2015; Delitto et al., 2018) and MyD88-independent pathways in mast cells and dendritic cells (Selander et al., 2009; Han et al., 2013). Notably, the activation of the MyD88-dependent pathway resulted in the activation of either MAPK or NF-κB pathways (Brown et al., 2011), both of which lead to the expression of functional products such as pro-inflammatory cytokines and chemokines (Akira and Takeda, 2004).

For pathogens, an important strategy of immune evasion is to bypass the host defensive response, for example by stimulating less cytokine secretion and lower immune cell activation, so as to increase tissue susceptibility to infection or other potential tissue insults (Nunes et al., 2013; Scumpia et al., 2017). This was also observed when RHG were exposed to the two pathogenic biofilms compared to the commensal biofilm. Similar to our finding, less activation on host immune response was found when gingival fibroblasts were exposed to the supernatant of a subgingival biofilm, which is more invasive and pathogenic compared to a supragingival biofilm. It was further suggested that NLRP3 and AIM2 inflammasomes and their down-stream IL-1 targets are involved in the mechanisms lead to the difference (Bostanci et al., 2011). Besides the upregulation induced by the commensal biofilm, we also found that gingivitis biofilm significantly up-regulated CD14; gingivitis and cariogenic biofilms both up-regulated IRAK2 transcription; while the cariogenic biofilm slightly suppressed CD14, IRAK4, and IRF3 transcription. The regulation of these genes may be related with some certain species in the biofilms. For example, the expression of CD14 was found up-regulated uniquely by a periodontal pathogen *Porphyromonas gingivalis* but not by *Streptococcus gordonii* or *Fusobacterium nucleatum* (Ebersole et al., 2019). Furthermore, we found that gingivitis biofilm in particular could activate CCL5 (MyD88-dependent and independent) transcription and cariogenic biofilm could activate CXCL10 (MyD88-independent) transcription. These results are in line with studies showing increased CCL5 in gingival cervical fluid in gingivitis and periodontitis patients (Gamonal et al., 2000; Gurkan et al., 2016), and another study showing the ability of caries-related bacteria to induce CXCL10 expression in dental pulp (Adachi et al., 2007).

*In vivo*, the healthy oral mucosa is not sterile but constantly exposed to abundant microbes. Therefore the host response to a new challenge is built on an already trained immune system, which may differ from *in vitro* (Novak et al., 2008). Here we simulated the initial host response by exposing the sterile RHG to multi-species biofilms. The commensal biofilm was able to prime the host by activating the TLR signaling pathway which results in inducing secretion of multiple functional cytokines, while the pathogenic biofilms showed less impact. In line with our findings, a study describing human neonatal cells being exposed to normal gastrointestinal flora showed that commensal bacteria triggered stronger innate immune responses in newborns than in adults. Furthermore, the increased pro-inflammatory activity was shown to be signaled via CD14, TLR2, and TLR4 (Karlsson et al., 2002). For oral mucosa, an increased immune response and immune cell filtration is considered necessary for policing the host barrier and eliminating pathogenic or noxious agents (Dutzan et al., 2016; Kosten et al., 2017), and can be induced not only by commensal microbes (Devine et al., 2015; Shang et al., 2018), but also by pure saliva (Cvikl et al., 2015). In patients with gingivitis or periodontitis, the periodontal tissue is typically more inflamed than in healthy people, which seems contrary to our finding. However, in these patients, the epithelial barrier is breached, the bacteria have entered the gingival soft tissues and tissue damage occurs. An immune response is triggered which results in inflammation, activation of osteoclasts or matrix metalloproteinase and damage to the alveolar bone and periodontal ligaments (Kinane et al., 2017). However, in our study we investigate the immediate tissue response to the biofilms over a 24 h period, where only limited tissue invasion and no tissue damage has occurred, and under these conditions, the pathogenic bacteria result in a much lower inflammatory response than the commensals.

As with all studies, in addition to its strengths, our model also has some limitations which should be noted. We performed parallel exposure of commensal or pathogenic biofilm for 24 h, although it would be more physiological related if we investigate the response of a commensal-primed RHG to pathogenic biofilms over an extended time period. However, such a setup is not desired due to extensive bacterial growth after 3 days of co-culturing without antibiotics. In the oral cavity, it can be expected that saliva and gingival crevicular fluid will influence the composition and the growth rate of the bacteria since it is continuously rinsing the gingiva and also contains many substances (mucus, enzymes, and antimicrobial compounds like immunoglobulin A), which are key factors influencing microbe behavior as well as the host response (Costalonga and Herzberg, 2014). Therefore a future challenge will be to also include saliva or a saliva substitute into the host-microbiome model. Another limitation of our model is the lack of key immune cells which are present in oral mucosa, such as neutrophils or dendritic cells, since these may affect our interpretation of host-microbe interactions via TLR signaling pathways. Therefore future studies should include our immune competent RHG which contains integrated Langerhans cells (Kosten et al., 2016). Also, the RHG model is specifically constructed in the way to mimic oral soft tissue. The absence of oral hard tissue (tooth or alveolar bone) may explain the less potent effects of cariogenic and gingivitis biofilm on the RHG, compared to the commensal biofilm which is generally distributed over the surface in oral cavity. A further limitation to acknowledge is the culture environment (biofilms were cultured anaerobically but applied to RHG aerobically), which may have resulted in a change in composition of the different biofilms compared to that previously reported (Janus et al., 2015). For those TLRs which recognize specific ligands, the activation effect may be partly diminished if the ligands within the cultured biofilm are not as abundant and active as *in vivo*, for example TLR9 (unmethylated CpG DNA), TLR3 (viral double-strand RNA), and TLR5 (bacterial flagellin from invading mobile bacteria). The strengths of our model are realized when considering its unique physiological relevance for investigating host-microbe interactions, compared to previous studies (Millhouse et al., 2014; Ramage et al., 2017; Herrero et al., 2018). The biofilms were cultured from human saliva in a way that mimics pathogenic transition from commensal to gingivitis or cariogenic biofilms, while most biofilms were constituted by up to 14 single species from *in vitro* cultures. There are more than 70 OTUs contained in our biofilms which correlates well to the fact that 30–300 species can be found in human oral microbiome. Compared to monolayer host cells, the organotypic RHG model is more representative for *in vivo*, and has already been used in studying the influence of external exposure on the host (Kosten et al., 2015, 2016; Buskermolen et al., 2016; Shang et al., 2018), therefore comparable results should be expected from present study. Our results are also in line with another *in vitro* study in which multi-species biofilm was shown to induce a higher cytokine gene- and protein-expression in epithelial cells than single-species biofilms (Ramage et al., 2017).

In conclusion, we investigated the influence of commensal and pathogenic multi-species biofilms on the host TLR signaling pathway in RHG. The activation of commensal biofilm on the transcriptions of TLRs and multiple downstream cascades, together with the increased functional products, indicated the important role of commensal bacteria in protecting the host. By keeping the innate immune response at a moderate level, homeostasis is maintained between the host and its microbial residents. In contrast, exposure to pathogenic biofilms (cariogenic and gingivitis) stimulated a response which might result in immune evasion thus enabling the pathogens to penetrate undetected into the host tissues.

## Data Availability

The datasets generated for this study can be found in the Gene Expression Omnibus https://www.ncbi. nlm.nih.gov/geo/query/acc.cgi?acc=GSE133422.

## Author Contributions

LS, DD, and SG designed the study. LS, DD, JB, MJ, SR, and SG contributed to acquisition, analysis, or interpretation of data. LS and SG drafted the manuscript. DD, MJ, BK, WC, and SG critically revised the manuscript for important intellectual content. All authors reviewed the manuscript and gave final approval.

### Conflict of Interest Statement

The authors declare that the research was conducted in the absence of any commercial or financial relationships that could be construed as a potential conflict of interest.
